# Transfer Accuracy of 3D-Printed Customized Devices in Digital Indirect Bonding: A Systematic Review and Meta-Analysis

**DOI:** 10.1155/2023/5103991

**Published:** 2023-09-16

**Authors:** Alessandra Campobasso, Giovanni Battista, Gianluigi Fiorillo, Giulia Caldara, Eleonora Lo Muzio, Domenico Ciavarella, Giorgio Gastaldi, Lorenzo Lo Muzio

**Affiliations:** ^1^Department of Clinical and Experimental Medicine, University of Foggia, Via Rovelli 50, Foggia 71122, Italy; ^2^Dental School, San Raffaele Vita-Salute University of Milan, Milan 20132, Italy; ^3^Department of Translational Medicine and for Romagna, University of Ferrara, Ferrara 44121, Italy

## Abstract

**Aim:**

To evaluate in vitro and in vivo the accuracy of 3D-printed customized transfer devices during indirect bonding technique (IBT).

**Methods:**

A search for articles published in the English language until April 2022 was carried out using PubMed, Web of Science, Scopus, and Google Scholar databases and by applying a specific search strategy for each database to identify all potentially relevant in vivo or in vitro studies. After the removal of duplicate articles and data extraction according to the participants-intervention-comparison-outcome-study design schema scheme, the methodological quality of the included studies was assessed using the Swedish Council on Technology Assessment in Health Care Criteria for Grading Assessed Studies.

**Results:**

The initial search identified 126 articles, 43 of which were selected by title and abstract. After full-text reading, 15 papers were selected for the qualitative analysis and seven studies for the quantitative analysis. The evidence quality for the selected studies was moderate.

**Conclusions:**

Except for the bucco-lingual direction, the 3D-printed customized devices have a transfer accuracy within the clinically acceptable limits established by the American Board of Orthodontics. Therefore, 3D-printed transfer devices may be considered an accurate method for bonding position during IBT, both in vitro and in vivo. Additional randomized clinical studies in vivo should be suggested.

## 1. Introduction

The accurate positioning of brackets is a critical factor in orthodontic treatment since the introduction of the straight-wire technique [1], as it can affect the results and duration of the overall therapy [2]. Therefore, achieving correct bracket positioning is crucial to attain the best possible outcome [3]. Orthodontic brackets can be placed on the tooth surface through either direct or indirect bonding methods. The direct bonding method consists of a one-step procedure, which involves the direct chair-side placement of brackets onto the enamel surface [4]. Although direct bonding is the most commonly used method, Silverman et al. [5] introduced the indirect bonding technique (IBT) in 1972. This technique was developed to optimize the accuracy of bracket positioning, as the ideal bracket positions are planned through a laboratory process in IBT [5]. In this technique, brackets were placed on dental casts and then bonded to the patients' teeth using a transfer device [6]. Therefore, the IBT allows for the reduction of clinical chair time and improvement in accuracy in bracket placement compared to the direct bonding technique [7]. In fact, the IBT decreases positioning errors due to clinical limitations such as low visibility, limited mouth opening, excessive salivary flow, or patient collaboration [8]. Moreover, during IBT, the reduced salivary contamination and limited chair time enable the containment of aerosol production and minimize personal contact, which are key factors in preventing contamination during orthodontic procedures [9, 10].

However, among the disadvantages of IBT, there are the difficulties of the inner technical procedures, which are related to the professional experience of the operator (including recording of alginate impressions and fabricating of dental casts and transfer trays). Moreover, IBT is associated with higher overall time and cost of laboratory procedures compared to the direct bonding technique [11, 12].

Computer-aided design and computer-aided manufacturing (CAD/CAM) technology have recently been introduced in orthodontic practice [2], and digital indirect bonding has emerged as a viable alternative to the traditional method, overpassing its complicated manual laboratory process with a digital workflow [13].

According to the literature, the use of an intraoral scanner and the virtual bracket setup has been shown to increase the accuracy of bracket positioning rather than using conventional impressions and manually placing brackets on plaster models, respectively [11].

However, bonding accuracy depends on the absence of discrepancies between the transferred position of orthodontic brackets and their virtual planned placement [14]. Therefore, the transfer devices are key factors in this process. In fact, in the traditional approach, the use of conventional materials could influence the bracket placement, reducing bonding accuracy during production, transfer, and removal of these transfer devices [15].

Actually, instead of conventional materials, clinicians may design and fabricate 3D-printed devices to transfer the virtual planned bracket position to the teeth [6].

In literature, different types of transfer devices have been proposed using 3D printing technology [16].

In addition to time and cost saving [12], the use of 3D-printed trays appears to reduce human error during laboratory steps, increasing the fit of the trays on teeth compared to traditional handwork and improving the precision of the indirect bonding [2].

However, the selection of 3D-printed materials and the design options of the tray are critical variables that may influence the accuracy of the digital indirect bonding [17].

In literature, a recent review conducted by Bakdach and Hadad [18] has analyzed the accuracy of 3D-printed transfer trays for indirect bonding, concluding that 3D-printed trays have an acceptable transfer accuracy. However, although this review [18] was published in March 2022, the articles search has been ended in August 2021, omitting a large number of studies conducted more recently [4, 11, 13, 17, 19–22].

Therefore, given the relevance of this topic and given the large amount of techniques currently available for tray design and 3D-printing, an update of the existing literature [18] needs to be done to upgrade the evidence-based efficacy of the IBT with 3D-printing technology.

## 2. Aim

Therefore, the aim of this systematic review was to evaluate the accuracy of 3D-printed transfer devices during IBT to answer the following questions:

- What is the accuracy of 3D-printed transfer devices in linear and angular measurements?
- Are there any differences between their design and 3D-printing characteristics?
- What is their accuracy in vivo?

## 3. Material and Methods

The present systematic review adhered to the PRISMA statement [23]. The protocol of this systematic review was preliminarily registered on PROSPERO (ID: CRD42022319757).

The study selection, the data collection, and the quality assessment were independently performed by two authors (A.C. and E.L.M.). Conflicts were resolved by discussion with a third author (L.L.M.). The level of agreement between the two reviewers was assessed using Cohen kappa statistics.

### 3.1. Eligibility Criteria

According to the participants-intervention-comparison-outcome-study design schema (PICOS), the inclusion and exclusion criteria are summarized in Table 1.

### 3.2. Information Sources and Search Strategy

The search for articles was carried out using four electronic databases (Table 2) and included publications in the English language until April 2022. A specific search strategy was developed for each database, as shown in Table 2. In addition, the reference and citation lists of the included trials and relevant reviews were manually searched.

### 3.3. Study Selection and Data Collection Process

All titles identified from the literature were screened and selected, following the inclusion and exclusion criteria. Duplicate studies were preliminarily excluded. The abstracts were examined, and the full texts of the remaining articles were assessed for eligibility before inclusion in the final analysis.

### 3.4. Data Items

The characteristics of the included studies (author, journal/year, study design, groups, type of transfer device, transfer design, initial teeth analyzed, final teeth analyzed, scanner, software for bracket positioning, software for tray design, 3D-printer, resin, bracket, bonding materials, software for superimposition, superimposition technique, main outcome, clinical relevance).

### 3.5. Methodological Quality Assessment

The Swedish Council on Technology Assessment in Health Care Criteria for Grading Assessed Studies (SBU) was used to evaluate the methodological quality of the studies included in this review [24]. The articles were categorized into three levels (A, B, and C) of evidence based on the following criteria:

- Grade A (high level of evidence): Randomized clinical study or prospective study that includes a well-defined control group, defined diagnosis and endpoints, and descriptions of diagnostic reliability tests and reproducibility tests.
- Grade B (moderate level of evidence): Same criteria as Grade A, except for blinding in outcome assessment. Cohort study or retrospective case series with a defined control or reference group, defined diagnosis and endpoints, and descriptions of diagnostic reliability tests and reproducibility tests.
- Grade C (low level of evidence): Articles that do not meet the criteria of Grade A or B, with large attrition, unclear diagnosis and endpoints, and poorly defined patient material.

After assigning a score to each study, the available evidence in the review was further classified into four grades:

1. Strong: at least two studies of level “A.”
2. Moderate: one study of level “A” and at least two studies of level “B.”
3. Limited: at least two studies of level “B.”
4. Scarce: fewer than two studies of level “B.”

### 3.6. Synthesis of Results

A meta-analysis was performed using Review Manager 5.4 (Copenhagen: The Nordic Cochrane Centre, The Cochrane Collaboration, 2011) to evaluate the transfer error of 3D-printed transfer tray in all dimensions (mesio-distal, occluso-gingival, bucco-lingual, torque, tip, and rotation). A random-effects model was used to accommodate heterogeneity across studies and mean differences with 95% confidence intervals and standard errors were considered for all evaluated outcomes. Statistical heterogeneity was assessed by the *χ*^2^-square test and the *I*^2^ index. When *I*^2^ was between 0% and 50%, the heterogeneity was considered low; when *I*^2^ was above 50%, the heterogeneity was defined as high.

## 4. Results

### 4.1. Study Selection

The initial search identified 126 articles from PubMed, Scopus, Web of Science, and Google Scholar. After removing duplicate studies and those that did not meet the eligibility criteria based on title and abstract, a total of 43 full-text articles were screened. Finally, a total of 15 papers were identified for the qualitative analysis according to the eligibility criteria. Seven studies were selected for the quantitative analysis.

**Figure 1:**
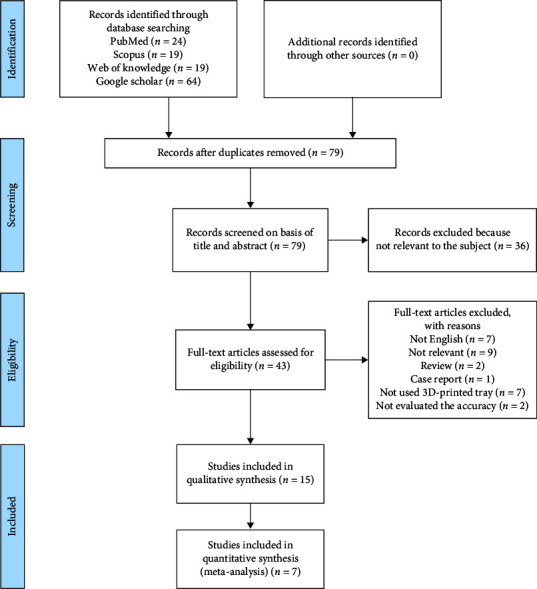
Flow diagram of the included studies according to the PRISMA.

The flowchart of the selection of eligible studies for this review is summarized in Figure 1.

### 4.2. Study Characteristics

The characteristics of the studies are presented in Tables 3 and 4. All studies were performed between 2017 and 2022. Three studies [16, 19, 25] were conducted in vivo, and the remaining 13 [2–4, 6, 7, 11, 13, 16, 17, 20–22, 26] were conducted in vitro, considering the transfer accuracy into mouth or dental models, respectively.

### 4.3. Assessment of Methodological Quality of Individual Studies

According to the SBU tool, the quality of evidence for one study [25] was high, and for two studies [16, 19] was moderate (Grade B). As a result, the level of evidence for the conclusions of this review was considered limited (level 3). The other included studies were laboratory studies.

### 4.4. Synthesis of Results

**Figure 2:**
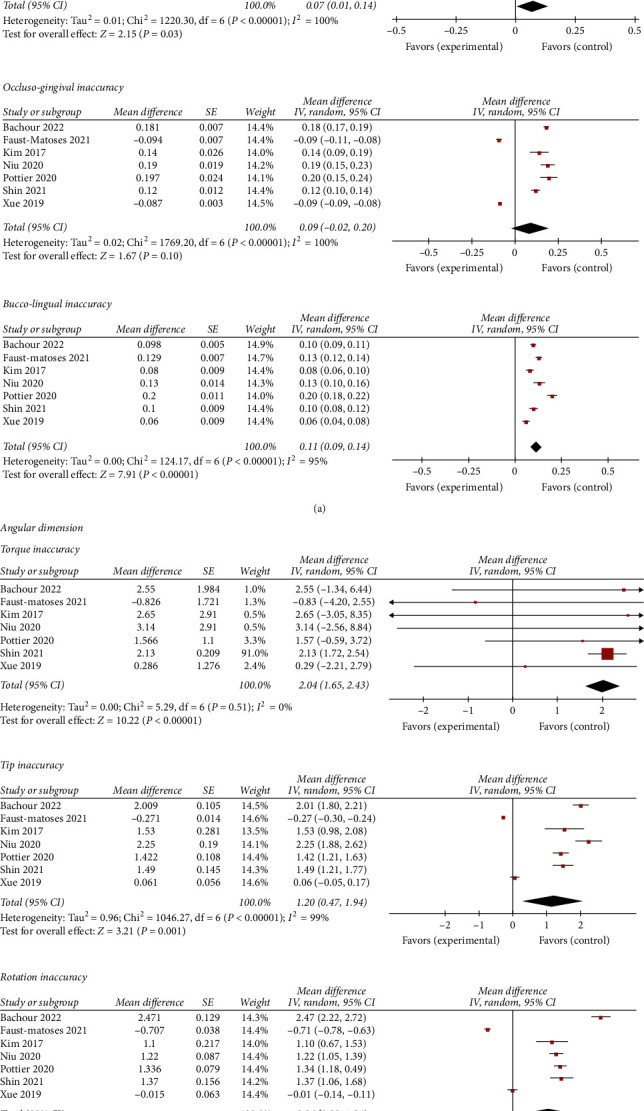
Forest plot comparing the transfer inaccuracy of the 3D-printed transfer trays among the studies (a) for linear measurements (mesio-distal, occluso-gingival, and bucco-lingual inaccuracies); (b) for angular measurements (torque, tip, and rotation inaccuracies). CI, confidence interval; IV, inverse variance; SE, standard error.

The linear (mesio-distal, occluso-gingival, and bucco-lingual) and the angular (torque, tip, and rotation) inaccuracy between the planned and the transferred bracket position were evaluated in all included studies (Figure 2), although only seven studies were included in the quantitative analysis [2–4, 6, 7, 16, 19] because the others were excluded due to lack of data or statistical analysis [13, 17, 20–22, 25–27].

#### 4.4.1. Transfer Inaccuracy for Linear Measurements

Mesio-distally, the mean transfer error was 0.07 mm (95% CI: 0.01, 0.14) with high heterogeneity (*χ*^2^=1220.30; *I*^2^ = 100%). Occluso-gingivally, the mean inaccuracy was 0.09 mm (95% CI: −0.02, 0.20) with high heterogeneity (*χ*^2^=1769.20; *I*^2^ = 100%). Bucco-lingually, the mean error was 0.11 mm (95% CI: 0.09, 0.14) with the lowest heterogeneity (*χ*^2^=124.17; *I*^2^ = 95%).

#### 4.4.2. Transfer Accuracy for Angular Measurements

The mean error in the torque was 2.04° (95% CI: 1.65, 2.43) with high heterogeneity (*χ*^2^=5.29; *I*^2^ = 0%). The tip inaccuracy was 1.20° (95% CI: 0.47, 1.94) with high heterogeneity (*χ*^2^=1046.27; *I*^2^ = 99%). The rotation error was 0.96° (95% CI: 0.09, 1.84) with high heterogeneity (*χ*^2^=1289.69; *I*^2^ = 100%).

#### 4.4.3. Transfer Accuracy In Vivo

**Figure 3:**
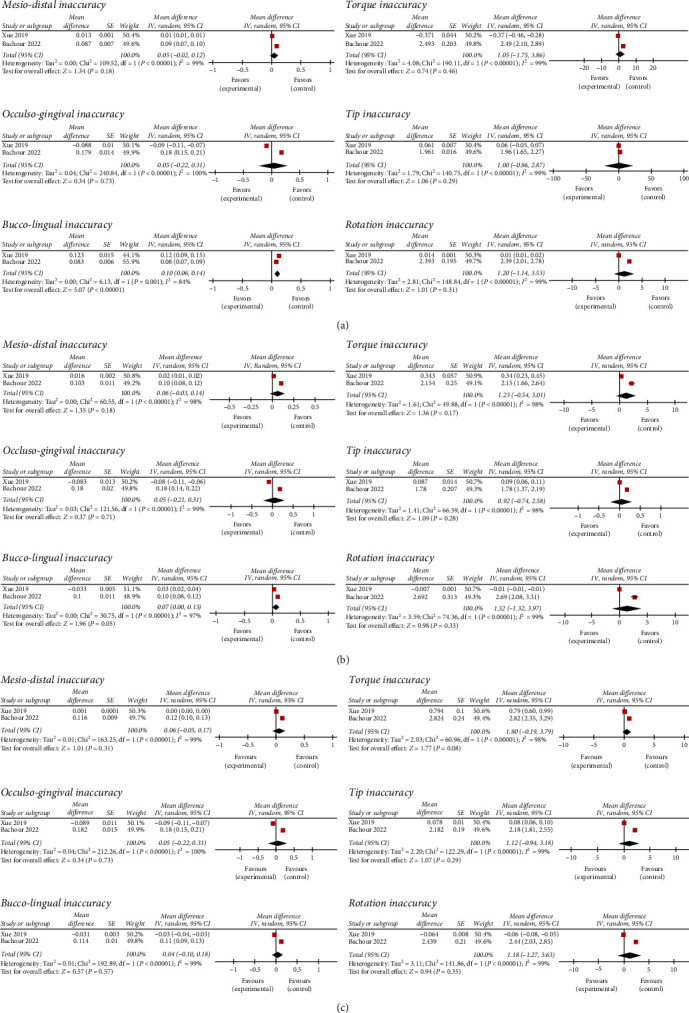
Forest plot comparing the transfer inaccuracy on of the 3D-printed transfer trays in vivo. (a) Transfer accuracy on incisors; (b) on canines; (c) on premolars. CI, confidence interval; IV, inverse variance; SE, standard error.

Among the three in vivo studies [16, 19, 25], only two studies [16, 19] were included in the quantitative synthesis according to the similar outcomes' measures (Figure 3). In the mesio-distal direction, incisor showed the lowest values of the inaccuracy of 0.05 mm (95% CI: −0.02, 0.12) (*I*^2^ = 99%), compared to the 0.6 mm reported both by canines (95% CI: −0.03, 0.14) (*I*^2^ = 98%) and premolars (95% CI: −0.05, 0.17) (*I*^2^ = 99%). In occluso-gingival direction, the mean transfer error was 0.05 mm for incisors (95% CI: −0.22, 0.31) (*I*^2^ = 100%), 0.05 mm for canines (95% CI: −0.21, 0.31) (*I*^2^ = 99%), and 0.05 mm for premolars (95% CI: −0.22, 0.31) (*I*^2^ = 100%). The highest bucco-lingual error was 1.0 mm, and it was reported by incisors (95% CI: 0.06, 0.14) (*I*^2^ = 84%), followed by 0.7 mm for canines (95% CI: 0.00, 0.13) (*I*^2^ = 97%), and by 0.04 for premolars (95% CI: −0.10, 0.18) (*I*^2^ = 99%).

Premolars showed the highest torque error of 1.80° (95% CI: −0.19, 3.79) (*I*^2^ = 98%), followed by canines with 1.23° (95% CI: −0.54, 3.01) (*I*^2^ = 99%) and by the incisors with 1.05° (95% CI: −1.75, 3.86) (*I*^2^ = 99%). The greatest tip inaccuracy was 1.2° for premolars (95% CI: −0.94, 3.18) (*I*^2^ = 99%), followed by 1.0° for incisors (95% CI: −0.86, 2.87) (*I*^2^ = 99%) and by 0.92° for canines (95% CI: −0.74, 2.58) (*I*^2^ = 98%). The mean rotation error was 1.20 at incisor level (95% CI: −1.14, 3.53) (*I*^2^ = 99%), of 1.32° (95% CI: −1.32, 3.97) (*I*^2^ = 99%) at canine level, and of 1.18° (95% CI: −1.27, 3.63) (*I*^2^ = 99%) at premolars level.

#### 4.4.4. Fi-Index Tool

This manuscript has been checked with the Fi-index tool and obtained a score of 0 for the first author only on April 17, 2023, according to SCOPUS® [28, 29]. The fi-index tool aims to ensure the quality of the reference list and limit any auto citations.

## 5. Discussion

### 5.1. Summary of Evidence

The aim of this systematic review was to evaluate the accuracy of the bonding position during IBT with 3D-printed transfer devices by assessing the linear and angular discrepancies between the transferred position of orthodontic brackets and their virtual planned placement.

Despite the satisfactory results achieved with the digital indirect approach, over the years, several CAD/CAM indirect bonding systems have been introduced [16, 18], and a range of transfer devices based on digital methods have become available to clinicians [2, 4, 11, 13, 16, 17, 19–21].

Transfer trays can be produced by 3D-printing a model with virtual planned brackets, using either traditional materials like silicone or thermoplastics, or by designing and directly manufacturing them through a 3D-printing process [16].

Many in vitro studies have compared the accuracy of 3D-printed trays produced with CAD/CAM technology to traditional systems [3, 6, 7, 22, 25, 26]. Chaudary et al. [25] observed greater accuracy with 3D-printed trays compared to silicone devices, except in the vertical dimension. Pottier et al. [7] also reported that, although silicone trays showed higher precision, both digital and conventional methods were considered clinically acceptable.

Both 3D-printed devices and vacuum-formed trays demonstrated comparable precision according to several studies [3, 6, 22, 26]. However, Zhang et al. [22] found that the conventional approach had the advantages of shorter fabrication time and lower costs.

The development of CAD/CAM software has simplified the virtual planning of the bracket position on digital casts, as well as the design and fabrication of customized 3D-printed devices to transfer the virtual planned bracket position from the 3D software to the teeth [6], making the indirect bonding procedure less reliant on operator experience [2].

According to Rattanasumawong et al. [26], using a digital workflow for designing and 3D-printing transfer trays not only saves time and costs [12] but also reduces the occurrence of human error during laboratory procedures, resulting in a superior fit of trays on teeth compared to conventional manual techniques and enhancing the precision of IBT [2].

Advancements in technology have significantly improved the precision of methods used to measure the accuracy of IBT, mainly due to the increased sensitivity of modern scanners and upgraded 3D software that enables virtual bracket positioning, tray design, and 3D image superimposition [7].

However, the accuracy of 3D superimposition programs can also be influenced by the technique used [18].

In the past, analyzing positioning errors relied on 2D photographs, which had limitations due to the operator's sensitivity [2]. Nowadays, 3D superimposition has replaced the 2D approach, and a wide range of 3D techniques are available to assess bracket positioning accuracy using 3D-printed transfer trays [7]. Clinicians typically use the local best-fit algorithm to superimpose specific dental surfaces with corresponding brackets [2, 4, 6, 7, 11, 13, 16, 17, 19–22, 26] or excluding brackets [3, 25], but transfer errors may occur if there is insufficient data in digital scans, such as in the contact surfaces between misaligned teeth [18].

Some studies have used digital bracket templates to superimpose scanned brackets [3, 16], but this method was less accurate due to the error within the method itself, as the surfaces of the matched brackets were not identical, and this led to inaccuracies in the calculation.

In addition to the increased accuracy of the overall 3D-printing workflow technique, other factors that may affect the accuracy of 3D-printed transfer trays include the type of printer and 3D-printed materials selected, tray design options, and clinical bonding procedures [17, 30].

Different 3D printing technologies are available for manufacturing 3D-printed devices, including stereolithography (SLA), the photo jet process, and digital light processing (DLP) [31].

SLA uses a laser to cure a light-sensitive polymer layer by layer in a vat of liquid polymer, while the photo jet process uses an inkjet print head to jet a light-sensitive polymer onto a build platform and cure it layer by layer. DLP cures liquid resin layer by layer using a projector light source, building the object upside down on a progressive elevating platform [31].

Several studies suggest that the positioning of dental models on the build platform of a 3D printer can affect the precision of the 3D-printed object [32]. As a result, various ideal printing directions have been proposed over the years. Hada et al. [33] and Unkovskiy et al. [34] reported that an angulation of 45° is the best orientation, while Shim et al. [35] identified 90° as the orientation for the most accurate manufacturing. Süpple et al. [32] reported that using DLP printers, the printing orientation on the build platform did not significantly affect the transfer accuracy.

Zhang et al. [36] found that for DLP technology, the optimal layer thickness was 50 *μ*m, although high printing accuracy was also achieved with a layer thickness of 100 *μ*m. They also found that for SLA technology, the printing accuracy increased with decreasing layer thickness.

Additionally, Arnold et al. [37] demonstrated that the placement of objects on the build platform of SLA printers can affect accuracy. They reported that the most accurate object was printed in the front of the platform, which contrasts with Unkovskiy et al. [34], who found that objects placed in the center of the build platform were more accurate than those placed at the border.

The rigid-flexible characteristics of the 3D-printed materials can also affect the accuracy of transfer trays. Only Hoffman et al. [17] compared the accuracy of two types of 3D-printable resins, reporting better accuracy with Dreve resin compared to Next Dent resin. They concluded that transfer errors can be reduced by using an appropriate 3D-printing tray.

Jungbauer et al. [20] demonstrated in vitro that the hardness of the printing materials can affect the bracket transfer accuracy, particularly in angular measurements.

While hard materials can result in an incomplete fit of the tray on dental surfaces or immediate bracket debonding during tray removal [12, 38], elastic characteristics can cause a tray distortion due to the pressure of the clinician's fingers, leading to overseating of the transfer devices and bracket positioning errors [8]. Therefore, it is important to design the parts of the tray that cover the brackets with the exact dimensions of each bracket to ensure appropriate retention during transfer and precise control during tray positioning [6, 7, 17]. Additionally, the design should allow for easy removal after bracket bonding to minimize the risk of debonding during tray removal [6]. These considerations are supported by research conducted by various authors [6–8, 12, 17].

To overcome these limitations, several authors have suggested alternative tray designs, such as partially covering the dental surfaces [16, 20], incorporating different retention mechanisms (e.g., arm-like sleeves, pocket-shaped designs) [21] or customized resin bases [27], or using a double-layer design with as ofter inside layer and a harder outside layer [6].

As suggested by De Oliveira et al. [14], bonding errors can also occur during clinical bonding procedures. Variations in the thickness of the bonding materials may result in insufficient or excessive adhesive application, leading to inaccurate positioning of the brackets on the teeth [16]. However, the use of transparent printable resin allows for intraoral checking of the tray fit and enhances the penetration of curing light during brackets polymerization, ultimately reducing the incidence of bonding failures, which is one of the most frequently reported disadvantages of IBT in the literature [12, 39].

Among clinical variables, the size and the shape of the teeth may affect the accuracy of transfer trays [13]. As reported by Kim et al. [2], the cusp height of posterior teeth did not affect the accuracy of both linear and angular measurements, in contrast to Jungbauer et al. [20], in which torque errors were more frequent at crowded frontal teeth. In addition, the use of resin base for individual teeth did not increase the accuracy, as reported by Park et al. [13].

Among the studies included in this analysis, the accuracy of 3D-printed trays was evaluated either in vitro by model scans [2–4, 6, 7, 11, 17, 21, 22, 26, 27] or in vivo by intraoral scans [16, 19, 25]. A 3D superimposition software was used to quantify bracket positioning errors in a local coordinate system, measuring linear (mesio-distal, occluso-gingival, and bucco-lingual) and angular (torque, tip, rotation) differences between the virtual and transferred bracket positions.

The signs (positive or negative) of the values indicated the direction of bonding displacement in relation to the reference position of each coordinate [13, 23]. Positive values indicated errors such as mesial, buccal, or gingival deviation, or more lingual torque, distal angulation, or mesial rotation of teeth compared to the planned position.

The direction of bonding displacement was expressed through positive or negative values [16, 25]; for example, positive values indicated errors such as mesial, buccal, or gingival deviation, or more lingual torque, distal angulation, or mesial rotation of teeth compared to the planned position.

The results of the present study indicated that the overall mean transfer error was within an acceptable range. The higher inaccuracy was observed in the buccal direction (0.11°), followed by gingival (0.09 mm) and by mesial (0.07 mm) directions.

Regarding angular measurements, the highest transfer errors were 2.04° for lingual torque, 1.20° for the distal tip and 0.96° for mesial rotation.

Positioning errors may affect treatment goals, and the literature has reported that linear errors less than 0.5 mm and angular errors less than 2° are considered clinically acceptable. However, over this value, teeth alignment and positioning of marginal ridges could be negatively influenced, as established by the American Board of Orthodontics [40].

Among the included studies, only a few studies have evaluated the accuracy of virtual bonding in the oral cavity using 3D-printed transfer trays in vivo [16, 19, 25], while most of the available studies have been performed in vitro on experimental dental casts [2–4, 6, 7, 11, 17, 21, 22, 26, 27].

However, the intraoral scanning procedure has been associated with a higher number of errors compared to laboratory model scans due to interference from salivary and soft tissues and lower lighting conditions, especially in the posterior region [2].

The findings of the present meta-analysis showed similar mesio-distal and occluso-gingival accuracy for incisors (0.05, 0.05 mm), canines (0.06, 0.05 mm), and premolars (0.06, 0.05 mm). The highest transfer error was reported in the bucco-lingual direction for incisors (0.10 mm), followed by canines (0.07 mm) and by premolars (0.04 mm). Among the angular measurements, the highest inaccuracies in torque and tip were found for premolars (1.80° and 1.12°, respectively), while the greatest rotation error was reported for canines (1.32°).

The maximum linear inaccuracy of incisors in the bucco-lingual direction may be explained by an improper fit of the tray caused by either a lack or an excessive pressure applied on the tray during IBT, especially when the brackets are not completely covered within the tray to facilitate its removal after bonding procedures [18].

Futhermore, the maximum inaccuracy in torque and tip could be influenced by the amount of the adhesive at the base of brackets [25], as an increased adhesive thickness or incorrect pressure applied on the tray due to the reduced visual check of the tray fit in the posterior region may affect the accuracy of bonding positioning [2, 6, 16]. In addition, placement errors at the premolars could also be due to the lack of precision of transfer devices in terminal areas [7] or to errors during scan data acquisition or during the tray fabrication process, which could affect the accuracy of IBT.

However, except for the bucco-lingual direction, all linear and angular measurements were within clinically acceptable limits. Therefore, in vivo, the use of 3D-printed transfer devices may be used for accurate IBT in vivo.

## 6. Conclusions

According to the SBU tool, the present review may draw conclusions reflecting a limited level of evidence.

- 3D-printed customized devices can be considered an accurate system for bonding position during IBT in both linear and angular measurements, both in vivo and in vitro.
- In vitro, the highest transfer error was reported in the bucco-lingual direction for linear measurements and in torque for angular measurements.
- In vivo, the highest transfer inaccuracy was reported in the bucco-lingual direction at the incisor level (for linear measurements) and in torque and tip of premolars (for angular measurements). However, the transfer accuracy of 3D-printed customized transfer trays appears to be within the clinically acceptable limits reported by the American Board of Orthodontics, except in the bucco-lingual direction.

Additional randomized clinical studies should be conducted to further assess the in vivo accuracy (possibly using standardized adhesive thickness during bonding procedures) and to evaluate the reproducibility of the IBT with 3D-printed transfer devices by different clinicians with varying clinical experiences.

## Figures and Tables

**Table 1:** List of inclusion and exclusion criteria, according to PICOS schema.

Field	Inclusion	Exclusion
Patients	Dental casts or dental arches treated with digital indirect bonding for labial bracket positioning	(i) Dental casts or dental arches treated with direct bonding or with traditional indirect bonding (ii) Use of lingual appliances

Intervention (exposure)	Use of 3D-printed transfer device	(i) Use of traditional transfer device (ii) Use of 3D-printed transfer device but without specific descriptions of the materials and applied technique

Comparison	(A) No comparison (for the descriptive analysis of the accuracy of 3D-printed transfer device) (B) Digital transfer device vs. traditional transfer device (comparison between 3D-printed and conventional materials, *such as double-layer or single-layer silicone rubber guide*, *double-layer or single-layer thermoforming plastic guide*)	

Outcome	Accuracy of orthodontic bracket transfer, in terms of: (i) Linear (mesio-distal, bucco-lingual, vertical) measurements (ii) Angular (angulation, rotation, torque) measurements	No clear mention of the analysis method

Study design	Randomized clinical trials or nonrandomized, prospective, or retrospective cohort studies	Review, case report, case–control study

**Table 2:** Database, search strategies, and results.

Database	Search Strategy	Results
PubMed searched on April 1, 2022 via https://pubmed.ncbi.nlm.nih.gov	((Accuracy) AND (indirect) AND (digital OR CAD-CAM OR computer-aided design and manufacturing) AND (bonding OR bracket ^*∗*^ OR position ^*∗*^) AND (transfer))	24

Web Of Science searched on April 1, 2022 via https://www.webofscience.com	((Accuracy) AND (“indirect bonding”) AND (digital))	19

Scopus searched on April 1, 2022 via https://www.scopus.com	((Accuracy) AND (“indirect bonding”) AND (digital))	19

Google Scholar searched on April 1, 2022 via https://scholar.google.com	((Accuracy) AND (3D-printed OR printed) AND (“transfer tray ^*∗*^” OR “transfer jig ^*∗*^” OR “transfer device ^*∗*^”) AND (digital OR “CAD-CAM” OR “computer-aided design and manufacturing”) AND (“indirect bonding”)) Google Scholar 64 risultati	64

**Table 3:** Characteristics of the included studies (I).

Author	Journal/year	Study design	Groups	Type of transfer device	Transfer design	Initial teeth analyzed	Final teeth analyzed	Scanner
Pottier et al.	Clin Oral Invest March 2020	In vitro laboratory study	(1) 10 Maxillary models, (2) 10 Maxillary models (1.5–2.5)	(1) Silicone tray, (2) 3D-printed tray	(1) Transparent, addition-cross-linking PVS with a thickness of 5 mm, (2) Hard acrylic tray, fitting the contour of the bck by closely holding them in the apertures, that were joined on the vestibular surface by a cylinder 2 mm in diameter and on the palatal surface by an extension of the mucous support of 4 mm	200 teeth (1.5–2.5)	195 teeth (97 group 1, 98 group 2)	Intraoral scanner TRIOS 2 color 3Shape

Kim et al.	AJO-DO June 2017	In vitro laboratory study	(1) Control group (5 maxillary plaster models), (2) Experimental group (5 maxillary plaster models with different cusp height)	3D-printed jig	Shape and size of the customized jigs are modified according to individual teeth; the manufacture of the final jig was completed in laboratory	60 teeth (1.4–1.6,2.4–2.6)	n.s.	Model scanner 7 series, dental wings

Xue et al.	AJO-DO March 2019	In vivo cohort study	(1) 1:10 patients (6 males, 4 females)	3D-printed L-shaped guides	(1) An L-shaped guides, fitting the occlusal and distal edge of the tie-wings on the bck, (2) A splint, covering the occlusal surface completely or partially, (3) Connecting rods that join the other 2 parts	205 teeth no bck failure in 3 months	205 teeth no bck failure in 3 months	Intraoral scanner TRIOS, 3Shape

Zhang et al.	BMC Oral Health 2020	In vitro laboratory study	(1) Digital guide group, (2) Traditional guide group	(1) Two types of 3D-printed guides, (2) Double-layer guide plate	(1) 3D-printed guides as whole denture type and single tooth type, (2) 1 mm inner layer (soft film); 0.8- or 0.6-mm outer layer (hard film) (Erkoform-3D Thermoformer)	140 teeth	n.s.	Model scanner TRIOS standard 3Shape

Chaudary et al.	J Orthod 2020	In vivo prospective randomized clinical trial	(1) Digital tray group (15 patients), (2) Conventional tray group (15 patients)	(1) 3D-printed tray, (2) PVS tray	(1) n.s., (2) PVS tray was fabricated with both light-body and heavy-body putty silicone	600 teeth	6 patients dropped out (bck loss after IB)	Intraoral scanner TRIOS color-913 shade, 3Shape

Rattanasumawong et al.	Applied Mechanics and Materials April 2020	In vitro laboratory study	(1) Conventional tray (5 arch set), (2) Conventional tray (5 arch set), (3) Digital groups (5 arch set)	(1) Vacuum-pressed thermoplastic tray (0.5 mm thickness), (2) Vacuum-pressed thermoplastic tray (0.5 mm thickness) 3 : 3D-printed tray	(1) Bck containers type, (2) Bck markers type, (3) Elastic resin with transparent, soft silicone and urethane parts	300 bck (1.5–2.5)	n.s.	Model scanner D2000 3Shape

Park et al.	KJO June 2021	In vitro laboratory study	(1) Bck with resin base group, (2) Bck without resin base group	(1) 3D-printed jigs, (2) 3D-printed jigs	(1) One-piece jigs with customized resin base (manually added), (2) One-piece jigs without a customized resin base	240 anterior teeth (116 upper, 109 lower) 1.3–2.3	226	Medit T500, medit for scan of initial plaster models Trios3, 3Shape for scan of models with transferred bck position

Jungbauer et al.	Applied Sciences June 2021	In vitro laboratory study	(1) 10 Mandibular models (minor crowding), (2) 10 Mandibular models (with severe crowding)	Hard and soft tray	The base (1 mm) covers all the occlusal/incisal surfaces + half of the lingual + about 1/3 of the labial surfaces of teeth; the molds (0.6 mm) cover the occlusal part of the bck/tb, overlapping the slot by 0.04 mm	n.s.	n.s.	(1) OrthoX scan, dentaurum (2) Microcomputed tomography (micro-CT)

Faust-Matoses et al.	Journal of Personalized Medicine September 2021	In vitro laboratory study	(1) Digital group (17 maxillary models, 17 mandibular models)	Transfer template in acrylic resin	n.s.	340 teeth (1.5–2.5; 3.5–4.5)	335	Models designed by Midas FX+, Brunleys

Niu et al.	Angle Orthod September 2020	In vitro laboratory study	(1) Digital tray group, (2) Vacuum-formed tray group	(1) 3D-printed tray, (2) transparent double vacuum-formed tray	(1) Buccally, extending until below bck and tb; lingually, covering half of the lingual surface until the distal marginal ridge of the first molars; 1.5 mm thickness, (2) Inside soft layer (2 mm); outside hard layer (1.5 mm)	240 teeth	212 teeth (27 debonded, 1 inverted) 108 bck for 3D-printed group; 104 for vacuum-formed group	Trios, 3Shape

Shin et al.	International Journal of Computerized Dentistry 2021	In vitro laboratory study	(1) Digital tray group (10 models), (2) Conventional group (10 models)	(1) 3D-printed tray, (2) Vacuum-formed tray	Thermoplastic 1 mm tray, covering the occlusal surfaces of the teeth and the lateral and the inferior surfaces of bck	280 teeth	n.s.	(1) Opapix 3D scanner, KOD600 (2) Orapix (for initial models). (3) TRIOS 3 scanner, 3Shape (after IB)

Park et al.	Sensors September 2021	In vitro laboratory study	(1) Bck with resin base group, (2) Bck without resin base group	(1) 3D-printed jig, (2) 3D-printed jig	(1) One-body transfer jig, (2) One-body transfer jig with a customized resin base manually added	506 teeth	n.s.	(1) Medit T500, Medit, for scan of initial plaster model (2) Intraoral Trios 3, 3shape, for scan of models with transferred bck position

Hoffmann et al.	Angle Orthod January 2022	In vitro laboratory study	(1) Digital tray group (10 upper models, (2) Digital tray group (10 upper models), (3) Conventional tray group (10 upper models)	(1) 3D-printed Dreve tray, (2) 3D-printed Next-Dent tray, (3) PVS tray	Digital trays: 0.05-mm distance to the tooth crowns, thickness of 1.3 mm, and a 1.5-mm slot overlap in the bck region, with flat design of the occlusal region. conventional trays: PVS putty (Tresident 2000K; Schutz Dental)	360 teeth (from 1.6 to 2.6)	n.s.	(1) Model scanner S300 ARTI for scan of initial plaster models, (2) Industrial scanner ATOS 5 (GOM) for scan of models with transferred bck position

Bachour et al.	Angle Orthod January 2022	In vivo prospective	(1) Digital tray (23 patients)	3D-printed tray	n.s.	410 bck (incisors, canines, and premolars of both arches)	n.s.	Intraoral scanner iTero Element, Align Technology

Von Glasenapp et al.	Journal of Clinical Medicine February 2022	In vitro laboratory study	(1) Digital tray group (27 models), (2) Digital tray group (27 models)	(1) 3D-printed tray version 1, (2) 3D-printed tray version 2	(1) Arm-like sleeves protruding from the tray base (and reaching into horizontal and vertical bck slots) segmented into tooth groups with small bridges connecting each section (more flexible), (2) Pocket-shaped design, enclosing bck and tubes from all sides (except gingival surface)	1,490 teeth (1070 bck + 420 tubes)	n.s.	Intraoral scanner Trios 3 W, 3Shape February 2022

*Notes*. n.s., not specified; PVS, polyvinyl siloxane; bck, brackets; tb, tubes; IB, indirect bonding.

**Table 4:** Characteristics of the included studies (II).

Author	Software for bck position	Software for tray design	3D-printer	Resin	Bracket	Bonding materials	Software for superimposition	Superimposition technique	Main outcome	Clinical Relevance
Pottier et al.	OrthoAnalyzer, 3Shape	Appliance Design software, OrthoAnalyzer, 3Shape	FormLabs 1+ (stereolithography)	40 D shore hardness, class IIa (Anenke Laboratory)at high elasticity	Metal Mini Master Roth 0.022-inch slot (American Orthodontics)	Transbond XT, 3M (bonding composite) Transbond MIP, 3M (adhesive primer)	GOM Inspect software Version 8 SR1	Best fit on palatal surfaces of the teeth	Higher accuracy in linear and angular values for silicone tray	Both are clinically acceptable (silicone is more accurate)

Kim et al.	3Txer, Orapix	3Txer, Orapix	Projet HD 3000 Plus; 3D Systems (photojet)	n.s.	Metal Mini Diamond Twin 0.018-inch slot (Ormco) buccaltb (Ormco)	n.s.	Geomagic Verify program (3D Systems) for superimpositions Geomagic Design X and Cimatron (3D Systems) for accuracy analysis	Best fit on the occlusal, buccal, and mesiodistal views of each tooth	Differences in cusp height of maxillary posterior teeth not significantly influence the accuracy (both for the linear and angular measurements)	Cusp height should be considered when using digital indirect bonding (for the higher frequency of placement errors in posterior teeth with large cusps)

Xue et al.	OrthoAnalyzer, 3Shape	Freeform software, Geomagic, version 12.0			Ceramic Clarity Adhesive Coated Advanced bck edgewise 0.022-inch slot (3M) buccal tb (Shinye Orthodontic Products)	Trans bond Moisture Insensitive Primer (3 M)	Geomagic Studio 2013, version 2013, Geomagic	Superimposition on the same region of bck	The accuracy is below the clinically acceptable range, except for the 15.12% of bck with torque deviation over 2°	High positional accuracy in vivo

Zhang et al.	OrthoAnalyzer, 3Shape	n.s.	Project 3510 DP	n.s.	n.s.	Transbond XT light-curable adhesive, 3M	Mimics software for 3D printing guide group	n.s. Digital caliper for traditional group	No significant differences between whole denture and single tooth type among 3D-printed guides; comparable results between 3D printing group and double-layer guide plates group (0.6 mm)	More working time and cost for 3D-printed guides compared to traditional ones (0.6 mm)

Chaudaryet al.	OrthoAnalyzer, 3Shape	Appliance Design software, OrthoAnalyzer, 3Shape	3D Stratasys (Objet 500)	n.s.	Metallic Victory series 0.022-inch slot (3M)	Sondhi Rapid-Set IB Adhesive, 3M	GOM Inspect software (GOM GmbH)	Best fit of individual teeth (without bck)	3D printed trays are more accurate than PVS trays except for vertical dimension	Clincally acceptable transfer errors in vertical dimension with 3D-printed trays

Rattanasumawong et al.	OrthoAnalyzer, 3Shape	Appliance Design software, OrthoAnalyzer, 3Shape	Form2, Formlabs (stereolithography)	n.s.	Metal Mini Master Roth 0.022-inch slot (American Orthodontics)	Transbond XT, 3M	GOM Inspect Version 8 SR1, GOM GmbH	Local best fit	In all three methods, position errors were less than 0.31 mm and more than 0.08 mm	3D-printed trays may reduce laboratory time and positional errors risk

Park et al.	3Txer, Cenos Co.,	n.s.	ProJet MJP 3600, 3D Systems (polyjet-type)	n.s.	Ceramic self-ligating bckQuicK-lear (Forestadent)	Transbond XT Primer, 3M (bonding agent) Transbond XT Light cure Adhesive, 3M (resin adhesive)	RapidForm software 2006 (INUS Technology)	Best-fit algorithm	The most pronounced errors in the vertical dimension (toward occlusal surface); tendency to transfer bck toward the buccal and mesial direction	Clinically acceptable for anterior teeth

Jungbauer et al.	OnyxCeph, Lab software, Image instruments	OnyxCeph, Lab software	Objet30 Dental Prime, Stratasys (polyjet process) for hard tray (high-speed mode, 28 *µ*m resolution) MoonRay Printer, SprintRay for soft tray (digital light process, 100 micron resolution)	MED610 (shore D hardness 83–86; Stratasys) NextDentOrthoo IBT (shore A 85 hardness), NextDent B.V.	Discovery smart bck (Dentaurum) For teeth 3.5–4–5 buccal tbOrtho-Cast M series (Dentaurum) for teeth 36–37–46–47	Transbond XT adhesive Trasnbond XT primer	GOM Inspect 2018, GOM	Local best fit algorithm	Minor linear discrepancies; greater deviations in torque than angular ones, higher for hard compared to soft trays; more pronounced torque errors at crowded front teeth	The impact of hardness and crowding on bck transfer accuracy should be carefully considered, specifically in torque and angular orientation

Faust-Matoseset al.	OrthoAnalyzer, 3Shape	Appliance Design software, OrthoAnalyzer, 3Shape	ProJet 6000. 3D Systems	Ortho IBT, NextDent	Clarity Advanced, 3M	APC Flash-Free Adhesive, 3M	3D Geomagic Capture Wrap, 3D Systems	Customized iterative closest point algorithm	The greatest errors in torque	Clinically accurate

Niu et al.	OrthoAnalyzer, 3Shape	OrthoAnalyzer, 3Shape		E- IDB 3D printing raw materials	Standard torque DamonQbck (Ormco) Snaplink buccal tb Ormco)	Primer Grengloo (Ormco) resin glue Grengloo (Ormco)	GOM Inspect 2018 software (GOM)	Local best-fit function (on selected tooth)	Higher accuracy of 3D-printed tray compared to vacuum-formed tray. Both types of trays have better linear control of bck compared to the angular one	Additional factors that could results in greater errors should be evaluated in vivo

Shin et al.	3Txer CAD software, Orapix 2021	Appliance Design software, OrthoAnalyzer, 3Shape Laboratory Study	(1) Perfactory Vida, Envision TEC (digital light- processing technology), (2) Conventional group (10 models)	(1) VisiJet M3X, 3D Systems, (2) Vacuum-formed tray	Clippy-C (Tomy) 0.018-inch Roth prescription	TRIOS 3 scanner, 3Shape (after IB)	Geomagic control X 2017, 3D System	Best fit algorithm, superimposing occlusal, lingual, and buccal surfaces, excluding bck	Greater height errors for incisors and molars are found in thermoplastic trays, compared to 3D-printed trays	3D-printed tray has a similar accuracy compared to conventional vacuum-formed ones, with slightly greater bck height accuracy

Park et al.	3Txer software, CENOS		Projet MJP 3600, 3D Systmes	n.s.	Self-ligated metal bckBioQuick® 0.022-in Tweemac prescription (Forestadent), from central incisors to the second premolars double and single tb (3M) for first and second molars	TransbondTM XT Primer, 3M, bonding agent TransbondTM XT Light Cure Adhesive, 3M resin adhesive	Rapidform software 2006, INUS technology	Best-fitalgorithm	The presence or absence of a resin base not consistently affect the accuracy	Resin base fabrication might not be essential in ensuring high-accuracy of digital indirect bonding

Hoffmann et al.	n.s.	n.s. OnyxCeph3TM, OnyxCeph 3D Lab	Projet MJP 3600, 3D Systems D20 II, Rap- idshape (digital light processing system)	(1) DreveFotoDent ITB (DreveDentamid), (2) NextDent Ortho ITB (NextDent)	BioQuick (Forestadent) slot 0.022-inch self-ligating bck and buccal tb	RelyX Unicem 2 Automix (3M)	OnyxCeph 3D Lab software	Best-fit algorithm with over 10 visually identifiable anatomical points in a radius of 1.0 mm	3D-printed trays achieve comparable results with the PVS trays. NextDent appears to be inferior compared with PVS regarding the frequency of clinically acceptable errors, whereas Dreve was found to be equal	Errors may be reduced by using an appropriate 3D-printed transfer tray (Dreve)

Bachour et al.	OrthoAnalyzer, 3Shape			Fotodent IBT 385 nm biocompatibleresin (DreveDentamid)	Victory Series slot 022-inch (3M) Mini-Master Series slot 018-inch (American Orthodontics)	Assure Plus primer, Reliance Orthodontic Products Transbond XT light-curing adhesive (3M)	VisionX Compare software (VisionX)	Best-fit superimposition using an iterative closest point matching algorithm (to achieve surface feature-based)	High positional accuracy of 3D-printed trays in linear dimensions, while questions remain for angular dimensions	The frequency of bck transfer error is approximately the same for all tooth types

Von Glasenapp et al.	OnxyCeph3TM, Image Instruments	Appliance Designer, 3Shape	Carbon digital light synthesis printer, Carbon	Methacrylate-based material Imprimo LC IBT, SCHEU-DENTAL flexible (Shore D hardness 40) and transparent light-curing resin	Discovery smart/discovery pearl bck Ortho-CastTM M-Series 0.018 inch, Roth (Dentaurum) Ortho-Cast M-Series tb, 0.018 inch, Roth (Dentaurum)	Transbond XT, 3M Transbond XT Primer, 3M	Geomagic Control, 3D Systems Inc.	Automated best-fit alignment	The transfer accuracy of two trays design IS comparable (although their different retention mechanism)	Both design suitable for clinical practice

*Notes*. n.s., not specified; bck, brackets; tb, tubes.

## Data Availability

The datasets used and/or analyzed during the current study are available from the corresponding author upon reasonable request.

## Abbreviations

IBT: Indirect bonding technique
CAD/CAM: Computer-aided design and computer-aided manufacturing
SBU: Swedish Council on Technology Assessment in Health Care Criteria for Grading Assessed Studies
SLA: Stereolithography
DLP: Digital light processing
ABO: American Board of Orthodontics.

## Additional Points

*Limitations*. The potential limitations of this review include the high heterogeneity of the included studies and the relatively small number of in vivo studies. Another potential limitation could be the different methods used for quantitative analysis, which may have influenced the magnitude of the positional deviations measured for each tooth.
